# Giant Right Coronary Ostial Aneurysm in a Patient With Marfan Syndrome

**DOI:** 10.7759/cureus.13627

**Published:** 2021-03-01

**Authors:** Rachel Stein, Rebekah M Padilla, Gregory Wynn

**Affiliations:** 1 Radiology, University of Florida College of Medicine – Jacksonville, Jacksonville, USA

**Keywords:** coronary artery aneurysm, marfan syndrome, ostial aneurysm, aortic root replacement, imaging

## Abstract

Aortic root dilation and aortic insufficiency are prominent causes of morbidity in Marfan syndrome. These pathologies necessitate surgical repair, including aortic root and aortic valve replacement procedures, to improve prognosis. Coronary artery aneurysms, particularly giant coronary ostial aneurysms, are rare complications of these surgeries in the Marfan population. Due to the significant life-threatening sequelae of coronary artery aneurysms, it is imperative to bring attention regarding this complication to the radiologist assessing thoracic imaging in this patient population.

## Introduction

Coronary artery aneurysms (CAAs) are defined as the segment of the artery in which dilation is 1.5 times the diameter of the adjacent segment [1,2]. The incidence of patients undergoing coronary angiography who are incidentally found to have CAAs is 0.2%-4.9% [1-3]. There are several variations of CAAs including giant coronary artery aneurysms (GCAAs), coronary ostial aneurysms, true aneurysms, and pseudoaneurysms. Most CAAs are typically found to be <2cm [4]. While there is no clear definition of GCAAs, there has been a wide range of proposed terminology including aneurysms >20mm, >40mm, >50mm, four times the normal diameter, or >4cm in diameter; however, GCAAs reaching >4cm are exceedingly rare [2,4,5]. For the purposes of this article, we will refer to GCAAs as localized coronary artery dilations greater than 2cm.

GCAAs are also most often found incidentally, with an incidence of 0.02% in the general population [2]. It is imperative for the radiologist to identify CAAs as they carry the life-threatening risks of thrombosis, distal embolization, ischemia, myocardial infarction, and potential rupture [1,3]. GCAAs hold the same risks as CAAs but with a higher tendency for thrombus formation [4,6]. Atherosclerosis is the most common cause; however, it is known to arise in patients with vasculitides and connective tissue diseases, such as Kawasaki disease and Marfan syndrome, respectively [2,6,7]. Aortic root dilation is a major contributor to the morbidity and mortality of patients with Marfan syndrome [8,9]. Surgical repair is often necessary via the modified Bentall procedure and aortic valve-sparing root replacement, both of which markedly improve the prognosis in Marfan patients [8,10]. Given both the post-operative procedural risk and the risk of cystic medial necrosis, Marfan patients are at increased risk for developing GCAAs [11,12].

While reported in the literature, CAAs and GCAAs following aortic root procedures are a rare complication [2,3,6-8,11-13]. Herein we discuss a case report of a patient with Marfan syndrome who developed a giant right coronary osital aneurysm 18 years after undergoing the modified Bentall procedure.

## Case presentation

A 35-year-old male with a past medical history of Marfan syndrome and hypertension underwent aortic root replacement with a mechanical composite valve graft, 27mm Carbomedics valve, 18 years prior to presentation. The patient was being treated with warfarin for mechanical valve anticoagulation. He gave a family history significant for Marfan syndrome in both his brother and father. A 2.2cm giant right coronary ostial aneurysm was incidentally discovered two years prior to presentation on a CT pulmonary angiogram obtained for suspected pulmonary embolism in the setting of shortness of breath. At that time, the patient was aware of this finding, due to prior imaging at an outside hospital. Repeat CT pulmonary angiogram performed approximately 14 months later, for dyspnea on exertion, revealed an increase in the aneurysm diameter to 2.6cm.

**Figure 1:**
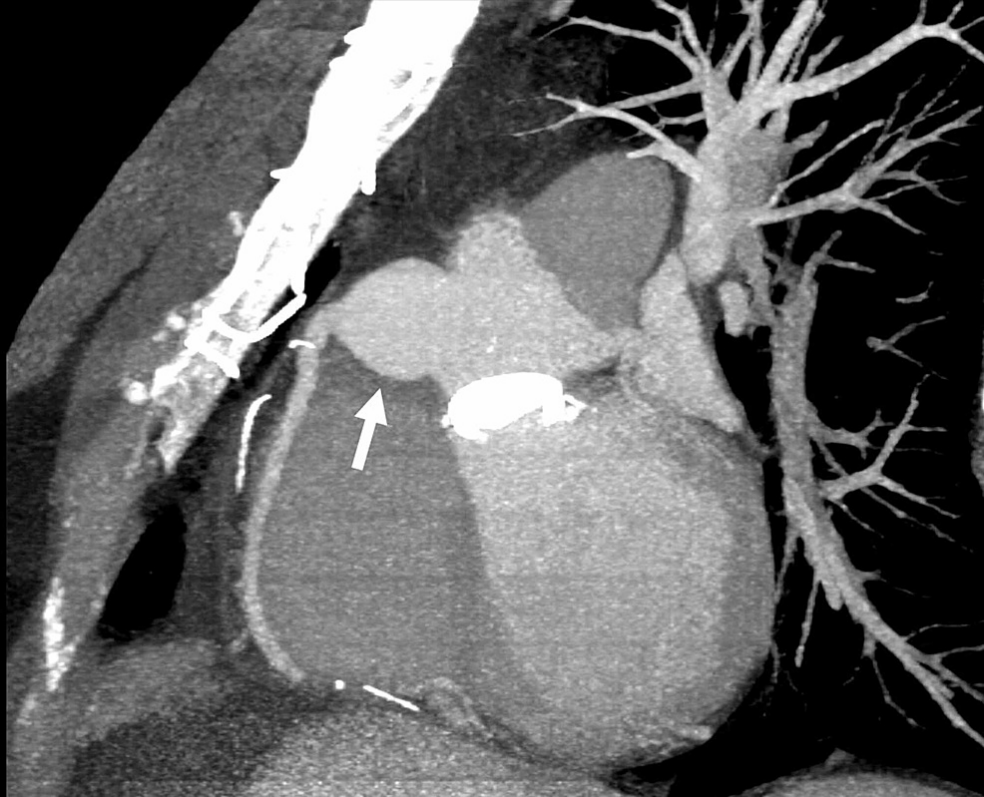
Maximum Intensity Projection Sagittal CTA Image Origin of the right coronary artery with aneurysmal dilatation of the first 3.5cm of the right coronary artery, which measures as much as 3.1cm in diameter (white arrow).

**Figure 2:**
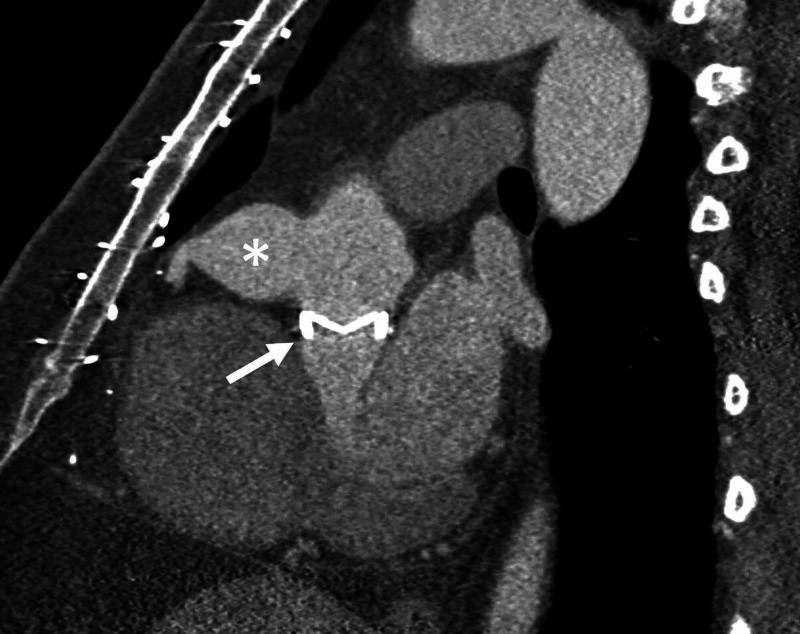
Multiplanar Reconstruction Sagittal CTA Image Prosthetic aortic valve replacement (Carbomedics 27 mm valve) (white arrow) and the aneurysm of the proximal right coronary artery and ostial dilatation (asterisk).

**Figure 3:**
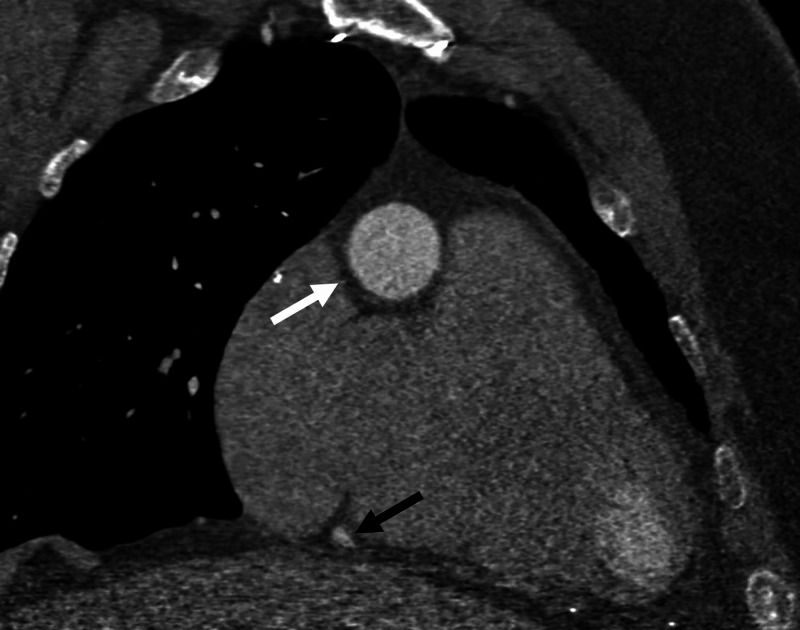
Multiplanar Reconstruction Oblique Coronal CTA Image Right coronary artery demonstrates aneurysmal dilatation of the artery just distal to the ostium (white arrow). The caliber of the distal right coronary artery is normal (black arrow).

**Figure 4:**
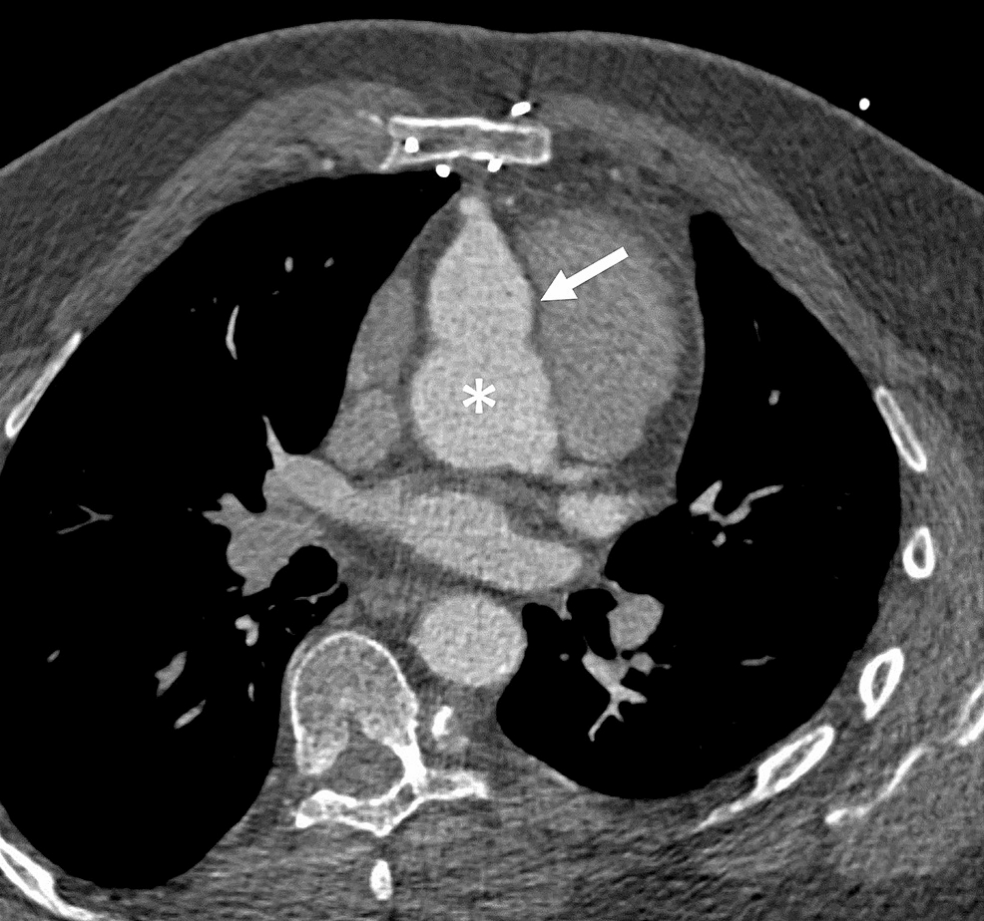
Multiplanar Reconstruction Axial CTA Image Ostial dilation and proximal aneurysm of the right coronary artery (white arrow) approaching the diameter of the proximal aorta (asterisk).

On this encounter, he presented for evaluation of the right GCAA, with the primary service requesting imaging as workup prior to surgical repair of this aneurysm. A CTA of the coronary arteries with retrospective electrocardiographic gating was performed on a Siemens 64 array multidetector computed tomography scanner (Siemens, Germany). The patient’s heart rate ranged from 58 to 75 beats per minute during the scan, with 0.4mg sublingual nitroglycerin given immediately prior to image acquisition for coronary artery vasodilation. Multiplanar reconstruction, dynamic loop-imaging, and maximum intensity projections were utilized. The images demonstrated the right GCAA was found to have increased to 3.1cm in diameter. The aneurysm originated from the right coronary artery ostium extending 3.5cm along the course of the right coronary artery (Figures 1-4).

## Discussion

Marfan syndrome is an autosomal dominant defect in fibrillin that causes a systemic disorder of connective tissue disease and is the most common genetic cause of aortic deficiency [3,8,9]. Aortic root dilation and aortic valve insufficiency account for the major morbidity and mortality associated with Marfan syndrome [9]. The modified Bentall procedure and aortic valve-sparing root surgery are two options for surgical management that have significantly improved the prognosis for Marfan patients with aortic root dilation [8,10,11,14]. Coronary ostial aneurysms are rare complications in Marfan patients following the modified Bentall procedure [2,3,6-8,11-13]. Knowledge of regional anatomy and the surgical technique utilized helps in understanding why these aneurysms occur in this patient population.

The aortic root connects the left ventricle to the ascending aorta and is comprised of the aortic annulus, sinuses of Valsalva, and sinotubular junction [15]. The coronary ostia arise from the dilated sinuses of Valsalva, which function to prevent occlusion of the ostia during systole and aortic valvular opening [16]. In both the modified Bentall procedure and aortic valve-sparing root surgery, the native coronary arteries are removed with a cuff of adventitia and re-implanted onto the aortic root graft, predisposing to wall weakness and possible aneurysm formation [8,13]. Marfan patients are also believed to be at increased risk for developing CAAs from cystic medial necrosis, which causes weakening of the tunica media in the vessel wall [2,12].

In the review of the literature, there are several types of CAAs that can occur after these procedures of which the radiologist should be aware. Definitions and measuring parameters for each type of aneurysm may be found in Table 1. As a radiologist, it is essential to understand the differences in aneurysm variations as treatment differs between them. The first step in defining the type of aneurysm is identifying whether the aneurysm is a true aneurysm or a pseudoaneurysm. On CTA, a true aneurysm will show contrast contained within the arterial lumen as compared to a pseudoaneurysm that demonstrates an extraluminal collection of contrast and may have a thin neck connecting the aneurysm to the involved coronary artery [8]. Most CAAs are <2cm in diameter [4]. Our patient presented with a 2.2cm aneurysm that progressed to 2.6cm over a 14-month period and further increased to 3.1cm over an additional 10 months. As alluded to earlier, there is wide variation in the definition of GCAAs; thus, we chose to label our patient’s aneurysm as a GCAA since it exceeded 2cm [4].

**Table 1:** Types of Coronary Artery Aneurysms and Their Definitions

Aneurysm	Definition
True aneurysm	Fusiform or saccular dilation of entire vessel wall
Pseudoaneurysm (false aneurysm)	Rupture or perforation of artery encased by blood clot or tunica adventitia
Coronary artery aneurysm	Localized dilation of 1.5 times or 50% more than the diameter of the adjacent segment
Giant coronary artery aneurysm	Aneurysm >2 cm to >4 cm in diameter
Coronary ostial aneurysm	>0.1 cm at the coronary ostial reimplantation site
Coronary button ostial aneurysm	>0.1 cm dilation of the “button” of native aorta at the ostia reimplantation site

While there is no clearly defined imaging protocol post-surgery, patients may undergo follow up with various imaging modalities, such as transthoracic ultrasound, CTA, magnetic resonance angiography (MRA), or coronary angiography [8]. Groner et al. state that CTA and MRA are the main surveillance imaging modalities of the post-surgical aorta in Marfan syndrome [8]. Electrocardiogram (ECG)-gated CTA has emerged to the forefront in assessing coronary artery anomalies and acute aortic diseases [17,18]. Earlier case reports of CAAs in Marfan patients post-surgery were diagnosed via non-gated CTA, whereas more recent reports, including our report, have diagnosed CAAs via ECG-gated CTA [7,11]. The benefit of ECG-gated over conventional non-gated CTA is the ability to decrease artifact by reducing aortic root motion, thereby improving the resolution of the coronary arteries and increasing diagnostic accuracy [11,18]. Often, invasive imaging with coronary angiography is recommended to further characterize the aneurysm’s size, shape, and specific location [2]. However, it has been suggested that ECG-gated CTA may be superior to coronary angiography [19].

Regardless of the imaging modality used, potential aneurysmal dilatation of the coronary arteries should be considered by the radiologist when assessing studies obtained in the Marfan population who have undergone previous aortic root procedures. Appreciation of the life-threatening risks of thrombosis, distal embolization, ischemia, myocardial infarction, and potential rupture associated with these aneurysms is also necessary [2,3]. Depending on the size of the coronary aneurysm, treatment modalities include medical management with anticoagulation, stenting, and coil embolization. Surgical repair is indicated if the aneurysm is three times the diameter of the original vessel [5]. Given the potential for a life-threatening rupture, GCAAs should receive surgical repair [12].

## Conclusions

Patients with Marfan syndrome are at increased risk of developing giant coronary ostial aneurysms following aortic root and valve replacement surgery. While no strict imaging protocol has been defined following these operative procedures in Marfan patients, ECG-gated CTA has emerged over time as the study of choice for coronary artery assessment. Defining GCAAs accurately is important as they carry a higher risk for thrombosis and necessitate surgical repair to prevent rupture.
